# *In Vivo* Bioactivities of *Hoya parasitica* (Wall.) and *In Silico* Study against Cyclooxygenase Enzymes

**DOI:** 10.1155/2022/1331758

**Published:** 2022-04-28

**Authors:** Kishore Kumar Sarkar, Trina Mitra, Md Asibur Rahman, Iqbal Mahmud Raja, Md Aktaruzzaman, Md Ahsan Abid, Md Nazmul Hasan Zilani, Debendra Nath Roy

**Affiliations:** Department of Pharmacy, Faculty of Biological Science and Technology, Jashore University of Science and Technology, Jashore 7408, Bangladesh

## Abstract

*Hoya parasitica* (Wall.) is extensively used in traditional medicine for the treatment of various diseases including rheumatism, kidney problems, jaundice, urinary tract disorders, fever, and pain. The present study was designed to explore new lead compound(s) to alleviate pain, pyresis, and diarrhea from methanol, ethyl acetate, and n-hexane extracts of *H. parasitica* (Wall.) leaves (MHP, EAHP, and NHP, respectively). Analgesic activity of the extracts was assessed through acetic acid induced writhing, tail immersion, and hot plate tests while brewer's yeast-induced pyrexia test was employed for the assessment of antipyretic activity. Besides, castor oil and magnesium sulfate induced diarrheal tests were utilized for the evaluation of antidiarrheal properties. Moreover, *in silico* study of the isolated compounds was undertaken to seek out best-fit phytoconstituent(s) against cyclooxygenase enzymes. MHP revealed substantial antioxidant activities in different *in vitro* assays compared to EAHP and NHP. In the acetic acid-induced writhing test, among the extracts, MHP (400 mg/kg) revealed maximum 74.15 ± 1% inhibition of writhing comparable to that of standard (85.77 ± 1.39%). Again, in tail immersion and hot plate tests, higher doses of all the test samples exhibited a significant increase of latent period in a time-dependent manner. In brewer yeast-induced pyrexia test, at 3^rd^ and 4^th^ hour of treatment, significant (*P* < 0.05) antipyretic action was found in the test samples. In both castor oil and magnesium induced diarrheal tests, MHP at 400 mg/kg showed the highest percent inhibition of diarrhea (68.62 ± 4.74 and 64.99 ± 2.90, respectively). Moreover, molecular docking analysis corroborated the results of the present study. The findings of the present study supported the traditional uses of this plant for the alleviation of pain and fever. Furthermore, hoyasterone was found to be the most effective lead compound as cyclooxygenase enzyme inhibitor.

## 1. Introduction

Revealing a lead compound that can limit the progression of an illness could be a crucial target to develop a potential new treatment plan over an existing one. Medicinal plants possess auspicious ways and means for devising novel healing agents. Numerous pharmacologically active compounds including alkaloids, glycosides, tannins, flavonoids, and volatile oils are noticed in medicinal plants. The current research trend is to look into plant-based medicines because they are more affordable and have fewer side effects [1].

Antioxidants are a variety of chemicals that are obtained from medicinal plants. Naturally, the human body produces free radicals. Excess free radicals react with various macromolecules such as DNA, lipid, and proteins, resulting in peroxidation and promoting the prognosis of aging, carcinogenesis, atherosclerosis, inflammatory disease, and cardiovascular disease. Antioxidants make an obstacle to the action of free radicals and prevent or halt these life-threatening diseases [2, 3]. Natural antioxidants are obtained from almost all human diets containing vegetables, fruits, and green plants according to the report of many researchers which play a vital role to reduce the risk of cancer, cardiovascular diseases, brain dysfunction, and cataracts [4]. Phenolic compounds work as hydrogen donors, reducing agents, and quenchers of singlet oxygen due to redox properties leading to antioxidant activities [5].

Pain is a common feature in human for which people seek treatment, is controlled by a complex web of interactions among molecular mediators and immune cells. Prostaglandins, histamine, interleukin-1, bradykinin, and platelet-activating factor seem to be the most commonly implicated mediators [6]. Pain and inflammation are currently treated with narcotics and non-narcotics corticosteroids [7]. Cyclooxygenase-1 (COX-1) and cyclooxygenase-2 (COX-2) are two distinct isoenzymes of cyclooxygenase (COX), which are required for the conversion of arachidonic acid into prostaglandins, prostacyclins, and thromboxanes. COX-1 is the primary mediator for maintaining stomach mucosal integrity, whereas COX-2 is mostly implicated in inflammation. The majority of NSAIDs suppress the activity of the cyclooxygenase enzyme in a nonselective manner, resulting in gastro intestinal ulcers, renal, cardiovascular, hematologic adverse events, and other side effects [8, 9]. Numerous phytochemicals obtained from a variety of medicinal plants were subjected for the assessment of antinociceptive as well as anti-inflammatory effects. For instance, different crude extracts including *Combretum molle* (combretaceae), *Millettia versicolor*, *Millettia griffoniana*, *Erythrina addisoniae*, and *Erythrina mildbraedii* together with their isolated compounds were found to reveal significant anti-inflammatory activity. In addition, *Pistacia integerrima* derived natural compound, pistagremic acid blocks COX-2 enzyme. Morphine, a narcotic alkaloid obtained from *Papaver somniferum*, results in alleviation of nociception and sedation acting on opioid receptor and ziconotide obtained from marine snail, *Conus magus*, elicits potential analgesic activity through blockage of N-type calcium channels which is a new nonopioid analgesic drug. Moreover, savinorin A derived from *Salvia divinorum* functions as a ƙ opioid receptor agonist whereas pawhuskin isolated from *Dalea purpurea* acts as an opioid receptor antagonist [10].

Diarrhea is the biggest cause of mortality, with over 1.8 million people dying from it each year [11, 12]. Children under the age of five are the most vulnerable group [13]. It is frequently a sign of an intestinal infection, which may be caused by a variety of bacteria, viruses, and parasites [14]. Mucosal injury, delayed healing of mucosal damage, and host susceptibility are all thought to be linked to chronic diarrhea, and all of them are impacted by diet [15]. Multiple studies report that malnutrition, lack of breastfeeding, infection, improper antibiotic usage, and living in an unsanitary environment are responsible for diarrhea disease [16, 17]. In general, diarrhea is commonly treated with antimotility and antisecretory medicines. Oral Rehydration Therapy (ORT) is used for treating diarrhea [18]. Pathogen-specific antidiarrheal drugs give proper ailments within a very short period of life [19].

Fever is a common feature of various pediatric diseases [20]. Pyrogens are the agents that cause fever. Exogenous pyrogens such as Gram-negative bacteria (endotoxins) and *Staphylococcus aureus* toxin produce fever. Endogenous pyrogens such as tumor necrosis factor-alpha (TNF-alpha), interleukin-1 (IL-1), interleukin-6 (IL-6), and other cytokines are stimulated by exogenous pyrogens [21]. Fever is provoked by increased prostaglandin E2 (PGE2) biosynthesis in the preoptic area of the hypothalamus that affects neuron firing rate, which is a major fever mediator [22]. Non-steroidal anti-inflammatory drugs (NSAIDs) such as ibuprofen, salicylates, and paracetamol are the most widely used drugs that act as antipyretic. With the substantial adverse effects of NSAIDs, there has been an increase in arguments over the judicious use of such medications [23].

*Hoya parasitica* Wall., an epiphyte that climbs and belongs to the family Asclepiadaceae, is frequently known as Waxflower or simply Hoya, Waxvine, Cherapata (Bengali), Pargacha, and Fassyagaas (tribal) [24]. It is a tropical evergreen shrub native to humid climates in South Asia, Australia, and Polynesia and tropical rain forests [25]. Moulvi Bazar, Sylhet, Chittagong, Satkhira, Sunderbans, Chittagong Hill Tracts, Cox's Bazar, and Jashore are among the places in Bangladesh where the plant may be found [26]. Previous study reported that triterpenic 3,4-seco acid 3,4-secolup-20 (29)-en-3-oic acid, as well as lupeolandlupenone, phenolics; 15-bulnesolic acid, 1-(4-hydroxy-3-methoxyphenyl)-1 methoxypropan-2-ol, androstanoid, and dihydrocanaric acid, sesquiterpene and triterpene compounds, have been isolated from *Hoya parasitica* [26, 27]. The leaf and fruit extract are used topically among the Chakma, Rem-Kalenga, and Tripura tribe to relieve limb pain, fever, jaundice, and constipation, respectively. In traditional medicine, this plant has been used as a treatment for diabetes, kidney diseases, bronchitis, urinary tract disorders, frequent or infrequent urination, rheumatic pain, paralysis, blood fever, body ache, antirheumatic, and acute renal failure [28]. Previous studies have reported the pharmacological uses of this plant in rheumatic, acute renal failure, jaundice, constipation, Alzheimer's disease, and anti-acetylcholinesterase and anti-butyryl cholinesterase activities [29–34]. Due to a few scientific attention on *Hoya parasitica* Wall., irrespective of its numerous traditional uses, it was selected to evaluate antidiarrheal, antipyretic, and antinociceptive activities. Furthermore, *in silico* study of the reported compounds of this plant was subjected on cyclooxygenase-1 (COX-1) and cyclooxygenase-2 (COX-2) to identify potential lead compounds which may be responsible for analgesic and antipyretic activities.

## 2. Materials and Methods

### 2.1. Collection and Authentication of Plant Material

*Hoya parasitic* leaves were collected from Sylhet, Bangladesh (at latitude 24°53′11.1696^″^ N and 91 52′50.5992^″^ E) and sent to the National Herbarium of Bangladesh, Mirpur-1, Dhaka, for its identification and the voucher Specimen (DACB-45169)was deposited in the Herbarium for further reference.

### 2.2. Preparation of Extracts

After being collected, the leaves were washed three times with flowing water and once with sterile distilled water and then dried at room temperature for a duration of seven days. Dried leaves were ground by a laboratory grinding mill (Model 2000 LAB Eriez®) and fine powder was obtained by passing through a 40-mesh sieve. Then, cold extraction was carried out by soaking 250 g of powdered material in 1.5 L of n-hexane, ethyl acetate, and methanol, respectively, in a order of lowest to highest polarity followed by continuous stiring and shaking. Filtration of the whole mixture was completed by clean sterilized cotton and filter paper (Whatman No. 1). Next, concentrated extracts were obtained by passing the filtrates in a rotary evaporator (Buchi Rotavapor Model R-124) under vacuum distillation and finally dried by desiccators to get 3.6 g, 4.8 g, and 6.9 g extracts, namely, n-hexane extract of *Hoya parasitica* (Wall.), ethyl acetate extract of *Hoya parasitica* (Wall.), and methanol extract of *Hoya parasitica* (Wall.) (NHP, EAHP, and MHP, respectively) having percent yield value of 1.44%, 1.92%, and 2.76% (w/w).

### 2.3. Experimental Animal

Male or female young Swiss albino mice with 6–7 weeks old (average) and a weight of 25–30 g were obtained from the Department of Pharmacy, Jahangirnagar University, Savar, Dhaka, Bangladesh. These experimental animals were housed in acceptable housing conditions with a relative humidity of 55–65% and a 12 : 12 h (light: dark) cycle, as well as a temperature of (27.0 ± 1.0) °C. The mice were fed rodent meals and given free access to water. For the adaptation of the experimental animals to the laboratory condition, a week was considered. The Ethical Review Committee, Faculty of Biological Science and Technology, Jashore University of Science and Technology authorized the *in vivo* experiments before the study and all of the experiments were carried out according to their standards [ERC/FBS/JUST/2018-15].

### 2.4. Experimental Design

Each *in vivo* pharmacological study was conducted using forty mice of either sex that were randomly selected and divided into eight groups of five mice in each. All groups were provided test materials orally as follows.

Group I (control): distilled water (10 mL/kg BW)

Group II: Standard (10/25/100 mg/kg BW)

Group III: MHP (200 mg/kg BW)

Group IV: MHP (400 mg/kg BW)

Group V: EAHP (200 mg/kg BW)

Group VI: EAHP (400 mg/kg BW)

Group VII: NHP (200 mg/kg BW)

Group VIII: NHP (400 mg/kg BW)

### 2.5. Chemicals and Reagents

Acetic acid, methanol, ethyl acetate, n-hexane, magnesium sulfate, and dimethyl sulfoxide (DMSO) were collected from Merck, Germany. Tween-80 and castor oil were obtained by Loba Chemie Pvt. Ltd., India. Moreover, diclofenac sodium and tramadol were bought from Square Pharmaceuticals Ltd. whereas loperamide HCl and paracetamol were from Beximco Pharmaceuticals Ltd. The solvents utilized in the experiments were of analytical grade.

### 2.6. Qualitative Phytochemical Screening

To detect the presence of phytochemical groups such as phenolic compounds, carbohydrates, alkaloids, saponins, acidic compounds, tannins, flavonoids, anthraquinones, glycosides, and gums, several qualitative tests were performed on freshly prepared MHP, EAHP, and NHP extracts through different color changes [28].

### 2.7. Acute Toxicity Study

In acute toxicity study, LD_50_ (half lethal dose) was measured for experimental samples in accordance with the guidelines of OECD (Organization of Economic Cooperation and Development) [35]. Grouping of the mice was categorized as control and test groups (NHP, EAHP, and MHP, respectively) having five mice in each. Individual mouse of the respective group was subjected to oral administration of test samples at the doses of 100, 250, 500, 1000, 2000, 3000, and 4000 mg/kg body weight and noticed for a duration of 14 days for any sign of toxicity through some parameters such as mortality, salivation, noisy breathing, food or water refusal, diarrhea, discharge from eyes and ears, injury, aggressiveness, changes in locomotor activity, pain, weakness, and coma [36].

### 2.8. Antioxidant Activity Study

#### 2.8.1. Total Phenolic Content Assay

Using the Folin-Ciocalteu (FC) reagent, the total phenolic content (TPC) of the extracts was determined spectrophotometrically [37]. To create the standard calibration curve, gallic acid was used as a standard, and various concentrations (0.1 to 0.5 mg/mL) were prepared. 1 mL of solution from each concentration was transferred into volumetric flasks followed by the addition of 9 mL distilled water and 1 mL FC reagent (10% v/v). After 5 minutes, 10 mL solution of sodium carbonate (7% w/v) was added and the volume was adjusted to 25 mL with distilled water. The absorbance was measured at 750 nm against a blank solution after 30 minutes of incubation. Using the calibration curve, the TPC of plant extracts was calculated as mg gallic acid equivalent per gram of dry extract.

#### 2.8.2. Total Flavonoid Content Assay

Using a standard quercetin calibration curve, the total flavonoid content (TFC) of MHP, EAHP, and NHP was measured [38]. For standard calibration curve, various concentrations of quercetin (0.1 to 0.5 mg/mL) were prepared. 1 mL of quercetin solution from each concentration was added to volumetric flasks followed by the addition of 4 mL of distilled water and 0.3 mL of sodium nitrous solution (5% w/v). Then, 0.3 mL of aluminum chloride (10% w/v) was added to the mixture after incubation of 5 min. After that, 2 mL sodium hydroxide (1 M) was added and adjusted the final volume to 10 ml. Next, the absorbance was measured against a blank at 510 nm. TFC of plant extracts was expressed as mg quercetin equivalent per gram of dry extract.

#### 2.8.3. Total Tannin Content Assay

The total tannin content (TTC) of fractionated extracts was determined using Folin-Ciocalteu's reagent [39]. To calculate TTC, 10 mg of extract was diluted in 10 mL of distilled water to yield a concentration of 1 mg/mL. To create the standard calibration curve, gallic acid at various concentrations (0.1 to 0.5 mg/mL) was made. In a summary, distilled water (7.5 mL) was blended with 0.5 mL of Folin-Ciocalteu's reagent followed by 0.1 mL extract solutions. The volume of the mixture was then adjusted to 10 mL by adding 1 mL sodium carbonate solution (35% w/v). Then, the absorbance was measured at 725 nm against a blank after 30 minutes of incubation. TTC of MHP, EAHP, and NHP was calculated as milligrams of gallic acid equivalent per gram of dried plant extract.

#### 2.8.4. DPPH Radical Scavenging Assay

DPPH free radical scavenging activity of the plant extracts was evaluated using the technique developed by Kumar et al. [40]. Various concentrations (1024, 512, 256, 128, 64, 32, 16, 8, 4, and 2 *μ*g/mL) of standard and extracts were obtained according to serial dilution technique. 3 mL of alcoholic DPPH solution (0.004%w/v) was added to each tube containing 1 mL of sample solution from each concentration. After 30 minutes of incubation at room temperature, the absorbance was measured at 517 nm using a UV-Visible spectrophotometer (Shimadzu, Japan). Ascorbic acid was employed as a standard. The following formula was used to compute the percent inhibition:

(1) $$\begin{array}{c} \%\text{Inhibition of DPPH}=\left(1–A_{1}/A_{0}\right)\times 100, \end{array}$$

where *A*_0_ is the absorbance of control and *A*_1_ is the absorbance of sample or standard.

#### 2.8.5. Reducing Power Assay

The reducing power assay was carried out according to the method narrated by Debnath et al. [41]. Serial dilution technique was followed to prepare different concentrations (500, 250, 125, 62.5, 31.25, and 15.62 *μ*g/mL) of standard as well as extracts. With regular shaking, 1 mL of an aliquot from each concentration was mixed with 2.5 mL of phosphate buffer (200 mmol/L, pH 6.6) and 2.5 mL of potassium ferricyanide (1% w/v). Next, 2.5 mL of 10 (w/v) trichloroacetic acid was added to the mixture after an incubation period of 20 minutes and then the mixture was centrifuged for 10 minutes at 3000 rpm. After that, 2.5 mL supernatant was collected and mixed with 0.5 mL ferric chloride (0.1% w/v) followed by continuous shaking. The absorbance was measured at 700 nm against a blank solution after 5 minutes.

### 2.9. Analgesic Activity Study

#### 2.9.1. Acetic Acid-Induced Writhing Test

The acetic acid-induced writhing test was performed according to the method of Zihad et al. [42]. Randomly forty mice were chosen and placed into eight groups, each with five mice: control (distilled water, 10 mL/kg body weight), positive control (diclofenac sodium, 25 mg/kg body weight), and test groups (MHP, EAHP, and NHP 200 mg/kg and 400 mg/kg body weight, respectively). Before the trial, the mice were fasted for 16 hours and given free access to water. After 30 minutes of oral treatment, each mouse of different groups was administered intraperitoneal injection of acetic acid (0.6% v/v) at a dose of 10 ml/kg body weight. Then, following 5 minutes interval, the number of writhes of each mouse was recorded for 10 minutes. The following formula was used to determine the percentage of inhibition of writhing:

(2) $$\begin{array}{c} \%\text{ inhibition of writhing}=\left(1-W_{0}/W_{1}\right)\times 100, \end{array}$$

where

*W*_1_ = the average number of the writhing of the control group.

*W*_0_ = the average number of the writhing of the standard or sample groups.

#### 2.9.2. Tail Immersion Test

This test was carried out according to the methods of Moushome et al. [36] and Khatun *et al.* [43]. This approach is based on the principle of extending the time necessary for mice to withdraw their tails from hot water containing morphine-like substances. When the tail tips of the mice were immersed in hot water (55 ± 1°C), the heat stimulation caused painful responses in the animals. The mice were grouped and the samples were administered in the same manner as in the aforementioned test. A cut-off point (15-second) was considered to prevent unintentional injury to the tails of mice. Mice of the positive control group were treated with tramadol (10 mg/kg). The latency response time (in seconds), i.e., the time necessary to withdraw from hot water, was calculated 30 minutes before the treatment of the corresponding group by immersing the mice's tail tips (last 1–2 cm) into hot water. The latency duration of tail-flick response was also measured at intervals of 30 minutes, 60 minutes, 120 minutes, and 180 minutes after each group receiving their respective treatment.

#### 2.9.3. Hot Plate Test

The hot plate test was performed using the Technique of Moushome et al. (2016) to assess the mechanism of central-acting analgesics [36, 44]. Before testing, mice were put on a hot plate maintained at 55 ± 1°C to be selected based on a cut-off point of 15 seconds. Before the trial, the mice were fasted for 16 hours and given free access to water. The mice grouping and sample administration were in the same manner described earlier. Besides, distilled water was provided to the mice of the control group at a dose of 10 ml/kg body weight and as well as tramadol (10 mg/kg) was utilized for positive control. Before 30 minutes of mice treatment, the latency response of the mice in each group (control, positive control, and MHP, EAHP, NHP 200 mg/kg and 400 mg/kg) was recorded. The latency response was also assessed at intervals of 30 minutes, 60 minutes, 120 minutes, and 180 minutes by observing various factors such as leaping, licking, and withdrawing the paw from the hot plate.

### 2.10. Antidiarrheal Activity Study

#### 2.10.1. Castor Oil Induced Diarrheal Test

The castor oil induced diarrheal test was carried out using the method developed by Tadesse et al. [45]. The mice were chosen for this experiment through the observation of diarrhea beginning after the administration of 0.5 ml of castor oil. Then, forty mice were split into eight groups including a control group (distilled water 10 mL/kg body weight), a positive control (loperamide HCl 3 mg/kg body weight), and test groups (MHP, EAHP, and NHP 200 mg/kg and 400 mg/kg, respectively) with five mice in each group. The mice were fasted for 16 hours before the experiment and given free access to water. Distilled water, loperamide HCL, and the test samples were administered orally as mentioned dose to the respective group of mice. After 30 minutes of the preceding treatments, each mouse was given 0.5 mL of castor oil orally to commence diarrhea. The mice were then placed in a separate cage with blotting paper at the bottom. For a period of 4 hours, the number of diarrheal feces was recorded and blotting sheets were replaced every hour. The percentage inhibition of defecation was calculated using the following formula:

(3) $$\begin{array}{c} \%\text{ inhibition of defecation}=\left(1-D_{1}/D_{0}\right)\times 100, \end{array}$$

*D*_0_ represents the mean number of defecations in the control group, whereas *D*_1_ represents the mean number of defecations in the test or standard group.

#### 2.10.2. Magnesium Sulfate Induced Diarrheal Test

The antidiarrheal effect of plant extracts was assessed using the MgSO_4_-induced diarrheal model proposed by Bello et al. [46]. The mice were chosen for the experiment based on the onset of diarrhea after receiving 2 g/kg of MgSO_4_ orally. The mice were starved for 16 hours but allowed to drink water freely. Mice were separated into eight groups and given the same treatment as described in the castor oil induced diarrheal test. After 30 minutes of pretreatments, the mice were administered magnesium sulfate at the dose of 2 g/kg body weight orally to induce diarrhea. For the positioning of each mouse, an isolated cage with blotting paper at the bottom was used. There was an observation period of 4 hours and during each hour the number of diarrheal feces was counted. The percentage inhibition of defecation was thus determined using the same formula employed in the castor oil-induced diarrheal model.

### 2.11. Antipyretic Activity Study

#### 2.11.1. Brewer's Yeast-Induced Pyrexia Test

This test was carried out following slightly modified method of Turner et al. [47]. Prior to the experiment, mice were starved overnight and given free access to water. Before producing pyrexia, their rectal temperatures were measured using an electronic thermometer attached to a probe and placed 2 cm into the rectum. Pyrexia was induced by injecting a 15% (w/v) brewer's yeast suspension into the back underneath the nape of the neck at a dose of 10 mL/kg body weight. By rubbing the injection site, the suspension was distributed beneath the skin. The rise in rectal temperature was measured 18 hours after injection, and mice with an increase in temperature of at least 0.6°C were deemed to be pyretic and eligible for the utilization in the brewer's yeast-induced pyrexia test. Then, the pyretic mice were administered test samples orally, which included distilled water (10 ml/kg) for the control group, paracetamol (100 mg/kg) for the positive control group, and plant extracts (MHP, EAHP, and NHP 200 mg/kg and 400 mg/kg) for the other test groups, respectively. Temperatures were measured at 1, 2, 3, and 4 hours for each group.

### 2.12. Statistical Analysis

All of the data were presented as mean ± SE (standard error). For the assessment of all test findings, a one-way ANOVA with Dunnett's test (*P* < 0.05, vs. control) was used. Furthermore, one-way ANOVA with post hoc Tukey's HSD test (*P* < 0.05, vs. standard/extract) was used to compare the mean values of the different groups (excluding control) which was regarded statistically significant. SPSS software was used to analyze all the data (version 20; IBM Corporation, New York, USA).

### 2.13. In Silico Study

#### 2.13.1. Ligand Preparation

To build a cumulative library of compounds of *H. parasitica*, data published about isolated compounds were collected from PubMed, Google Scholar, Web of Science, and Scopus by searching the key of “*Hoya parasitica*” and “isolated compounds.” From there, we found four new compounds, namely, hoyasterone (PubChem CID 101449264), 15-bulnesolic acid, 1-(4-hydroxy-3-methoxyphenyl)-1-methoxypropan-2-ol (PubChemCID 78152111), and dihydrocanaric acid (PubChem CID 101449266) [48, 49].

Those isolated compounds were selected for virtual screening to identify their potential analgesic and antipyretic activities against cyclooxygenase (COX) enzymes. After downloading those compounds from the PubChem database (https://pubchem.ncbi.nlm.nih.gov/) [50] their energy was minimized using GaussView 5.0.8 software package through B3LYP correlation function of the DFT method [51, 52].

#### 2.13.2. Protein Preparation

The 3D X-ray crystallographic structure of COX-1 (PDBID:3N8Y) and COX-2 (PDBID: 1PXX) was retrieved from Protein Data Bank (http://www.rcsb.org/) [53] in pdb format. Then, energy of those proteins was minimized through the implemented steepest descent and conjugate gradient technique of Swiss-PDB Viewer 4.10 [54]. By this process -31376.07 and -29203.19 kj/mol energy was minimized from 3N8Y and 1PXX, respectively, and computations were done in vacuo with GROMOS 96 43B1 parameters set without reaction field. After that, PyMOL DLP 3D [55] was used to remove water molecules and unnecessary residues from those crystal structures.

#### 2.13.3. Molecular Docking Analysis and Visualization

Virtual screening of the compounds against COX-1 and COX-2 was carried by Autodock 4.2.6 [56]. Energy of all compounds was further minimized through Universal Force Field (UFF) and the steepest descent optimization algorithm of this software. After that, input ligands were converted into pdbqt format by Python Molecular Viewer with AutoDock tools, required by AutoDockVina [57]. Then, all of the compounds were virtually screened at the active site of COX-1 and COX-2. The size of the grid box was kept 28.14, 40.23, and 36.24 Å dimensions for 3N8Y whereas 36.90, 30.90, and 38.77 Å dimensions for 1PXX along with the XYZ directions using Vina search space facilities. Binding affinity of each compound was chosen as a negative score and higher negative energy indicates better binding affinity. Then, using PyMOL [55] complexes of respective compounds and corresponding protein were formed and visualized to Discovery Studio 4.5 [58].

### 2.14. ADMET (Absorption, Distribution, Metabolism, Excretion, and Toxicity) and Drug-Likeliness of Ligands

In order to predict the pharmacokinetic profile, drug-likeliness, and medicinal chemistry friendliness of the experimental compounds of *H. parasitica*, SwissADME [59] and pkCSM [60] web tools were utilized where molecular weight (g/mol), number of hydrogen bond acceptor, number of hydrogen bond donor, octanol/water partition coefficient, number of rotatable bond, maximum tolerated dose for human, number of Lipinski's Violation, and drug likeness of the ligands were calculated.

## 3. Result

### 3.1. Qualitative Phytochemical Screening

Preliminary phytochemical screening revealed the presence of different phytochemical groups in NHP, EAHP, and MHP. All extracts contained carbohydrates, tannins, alkaloids, flavonoids, glycosides, and gums whereas steroids and anthraquinones were present in NHP but absent in EAHP and MHP. Moreover, phenolic and acidic compounds were absent in all extracts except NHP [Table 1].

### 3.2. Acute Toxicity Study

During the 14 days observation period, it was reported that even at the highest dose of 4000 mg/kg body weight caused no death, toxicity, or behavioral abnormalities. Moreover, the control group had the same experience. This means that the fatal doses of MHP, EAHP, and NHP needed to cause acute toxicity is greater than 4000 mg/kg.

### 3.3. Antioxidant Activity Study

#### 3.3.1. Total Phenolic Content Assay

In this assay, maximum amount of total phenolic compound was found to be 253.10 ± 1.56 mg GAE/g dry extract in MHP compared to that of EAHP and NHP (225.15 ± 0.51 and 79.70 ± 1.03 mg GAE/g dry extract, respectively) from gallic acid calibration curve (*Y* =0.996*X*+0.064; *R*^2^ = 0.982).

#### 3.3.2. Total Flavonoid Content Assay

In total flavonoid content assay, using calibration curve of Quercetin (*Y* =0.483*X* +0.014; *R*^2^ = 0.989), it is observed that MHP contained the highest amount of TFC (197.72 ± 1.03 mg QE/g dry extract) while for EAHP and NHP the amounts were (171.84 ± 2.07 and 104.55 ± 1.03 mg QE/g dry extract).

#### 3.3.3. Total Tannin Content Assay

MHP disclosed the presence of the highest amount of TTC (112.70 ± 1.16 mg GAE/g dry extract) compared to that of EAHP and NHP which was estimated from calibration curve of gallic acid (*Y* =1.291*X*+0.024; *R*^2^ = 0.980).

#### 3.3.4. DPPH Scavenging Assay

**Figure 1:**
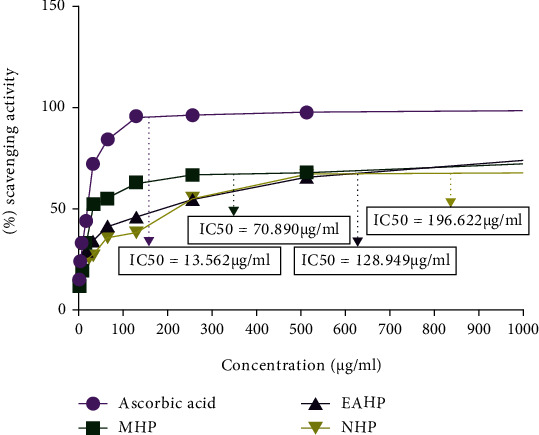
Comparison of DPPH scavenging activity among ascorbic acid, MHP, EAHP, and NHP.

In this assay, concentration-dependent DPPH scavenging activity was displayed by all extracts, namely, MHP, EAHP, and NHP, compared to that of ascorbic acid. Besides, IC_50_ values of MHP, EAHP, and NHP were 70.890, 128.949, and 196.622 *μ*g/mL, respectively, whereas standard showed IC_50_ value of 13.562 *μ*g/mL (Figure 1).

#### 3.3.5. Reducing Power Assay

**Figure 2:**
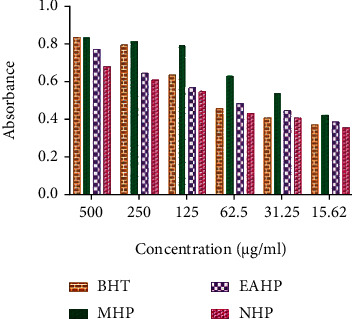
Comparison of reducing power among BHT, MHP, EAHP, and NHP.

All the extracts displayed reducing power in a concentration-dependent manner and the maximum absorbance was found to be 0.853 and 0.833 showed by the highest concentration (500 *μ*g/mL) of BHT and MHP, respectively (Figure 2).

### 3.4. Analgesic Activity Study

#### 3.4.1. Acetic Acid-Induced Writhing Test

In the acetic acid-induced writhing test, all the test samples (MHP, EAHP, and NHP, respectively) demonstrated significant percentage inhibition of writhing at the dose of 200 mg/kg and 400 mg/kg in comparison to control (*P* < 0.05). Moreover, higher dose (400 mg/kg) of MHP revealed 74.15 ± 1.00% inhibition of writhing which was maximum among all the groups and was comparable to that of standard (85.77 ± 1.39%) [Table 2].

#### 3.4.2. Tail Immersion Test

Higher doses of all the test samples (MHP, EAHP, and NHP, respectively) gradually exhibited a significant increase in latency period in a time-dependent manner except minor fluctuations (*P* < 0.05, vs. control). Moreover, MHP at the dose of 400 mg/kg significantly showed the maximum latency response at the 5^th^ observation period [Table 3].

#### 3.4.3. Hot Plate Test

After the administration of tramadol and all the test samples (MHP, EAHP, and NHP), the significant latency response was elicited in a time-dependent manner except minor fluctuations (^∗^*P* < 0.05, vs. control). Again, tramadol and higher dose (400 mg/kg) of MHP and EAHP showed a gradual increase in latency response from the 2^nd^ to 5^th^ observation period with maximum effect at 180 min compared to control (*P* < 0.05) [Table 4].

### 3.5. Antidiarrheal Activity Study

#### 3.5.1. Castor Oil Induced Diarrheal Test

**Figure 3:**
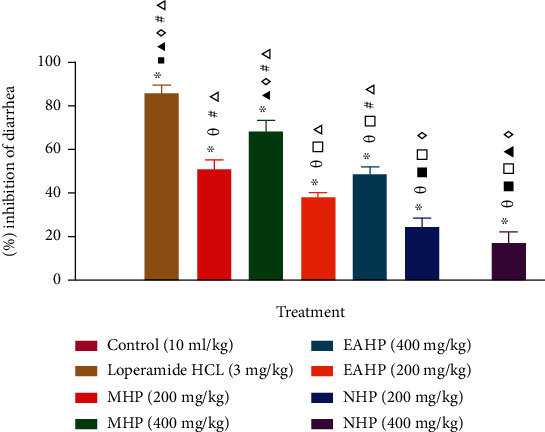
Effect of MHP, EAHP, and NHP in castor oil-induced diarrheal test. Values are presented as mean ± standard error of mean. *n* =5 mice in each group. ^∗^*P* < 0.05, versus control (Dunnett's t test); ^*θ*^*P* < 0.05, versus Loperamide 3 mg/kg; ^■^*P* < 0.05, versus MHP 200 mg/kg; ^□^*P* < 0.05, versus MHP 400 mg/kg; ^▲^*P* < 0.05, versus EAHP 200 mg/kg; ^◊^*P* < 0.05, versus EAHP 400 mg/kg; ^♯^*P* < 0.05, versus NHP 200 mg/kg; ^Δ^*P* < 0.05, versus NHP 400 mg/kg (pair-wise comparison by post hoc Tukey's HSD test).

In the castor oil induced diarrheal test, the lower and higher doses (200 mg/kg and 400 mg/kg) of all test samples (MHP, EAHP, and NHP) exhibited considerable percentage inhibition of diarrhea compared to the control (*P* < 0.05). However, MHP 400 mg/kg showed the highest percent inhibition of diarrhea (68.62 ± 4.74) comparable to that of standard (86.04 ± 3.75) demonstrating antidiarrheal effect [Figure 3].

#### 3.5.2. Magnesium Sulfate Induced Diarrheal Test

**Figure 4:**
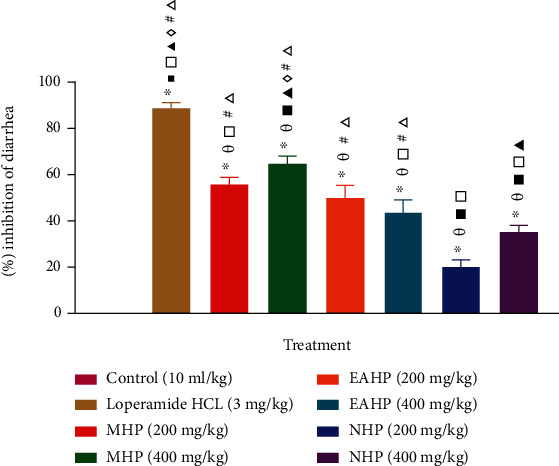
Effect of MHP, EAHP, and NHP in magnesium sulfate-induced diarrheal test. Values are presented as mean ± standard error of mean. *n* =5 mice in each group. ^∗^*P* < 0.05, versus control (Dunnett's t test); ^*θ*^*P* < 0.05, versus Loperamide 3 mg/kg; ^■^*P* <0.05, versus MHP 200 mg/kg; ^□^*P* < 0.05, versus MHP 400 mg/kg;^▲^*P* < 0.05, versus EAHP 200 mg/kg; ^◊^*P* < 0.05, versus EAHP 400 mg/kg; ^♯^*P* < 0.05, versus NHP 200 mg/kg; ^Δ^*P* < 0.05, versus NHP 400 mg/kg (pair-wise comparison by post hoc Tukey's HSD test).

Again, both the lower and higher doses (200 mg/kg and 400 mg/kg) of all test samples (MHP, EAHP, and NHP) demonstrated similar results in the magnesium sulfate induced diarrheal test where maximum percent inhibition of diarrhea was shown by MHP 400 mg/kg compared to standard Loperamide HCl (64.99 ± 2.90 and 88.67 ± 2.18, respectively) suggesting antidiarrheal property [Figure 4].

### 3.6. Antipyretic Activity Study

#### 3.6.1. Brewer Yeast-Induced Pyrexia Test

In brewer yeast-induced pyrexia test, during 3^rd^ and 4^th^ hour of treatment, significant antipyretic actions were demonstrated by all the test samples in comparison to control (*P* < 0.05). However, in comparison to paracetamol 100 mg/kg, MHP 400 mg/kg showed maximum antipyretic activity [Table 5].

### 3.7. In Silico Study

#### 3.7.1. Molecular Docking of Ligands with COX-1 Enzyme

**Figure 5:**
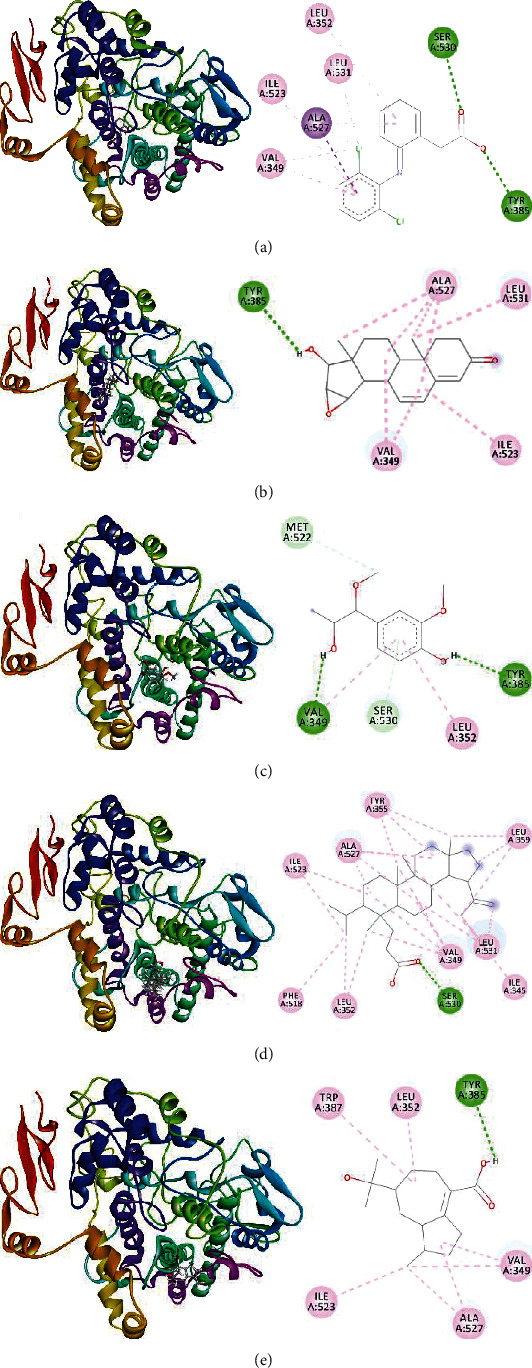
3D and 2D interaction of (a) diclofenac, (b) hoyasterone, (c) 1-(4-hydroxy-3-methoxyphenyl)-1-methoxypropan-2-ol, (d) dihydrocanaric acid, and (e) 15-bulnesolic acid, respectively, with COX-1 enzyme. Green, pink, light pink, and light green colors of the 2D image indicate hydrogen bond, Pi-Pi T shaped bond, Pi-alkyl bond, and carbon-hydrogen bond of that compound with COX-1.

Diclofenac is non-steroid benzene acetic acid consisting of phenylyacetic acid with a (2,6-dichlorophenyl) amino group at the 2-position. The carboxyl group of phenylyacetic acid moiety was chelated by SER-530 and TYR-385 through the formation of two different convention hydrogen bonds at 2.53 and 2.68 Å, respectively. Besides, this moiety formed two alkyl bonds with LEU352 and ALA527. On the other hand, (2,6-dichlorophenyl) amino moiety formed different types of hydrophobic bond with LEU531, ALA527, ILE523, and VAL349 (Table 6 and Figure 5 a). From the molecular docking, we found stable diclofenac forming different types of hydrogen and hydrophobic bonds at the inhibitory site of COX-1 along with binding affinity of -8.1 kcalmol^−1^.

From docking results, we observed that hoyasterone showed higher binding affinity of -8.5 kcalM-1 to COX-1. Hydroxyl group at C17 of hoyasterone formed a conventional hydrogen bond with the inhibitory motif TYR385 at a distance of 2.78 Å. Moreover, C18 formed two alkyl bonds with LEU531 and ALA527. On the other hand, among four rings of hoyasterone, the second ring formed three individual alkayl bonds with ILE523, VAL349, and ALA527 whereas the third ring form two alkyl bonds with VAL349 and ALA527, respectively. Additionally, C19 also interacted with ALA527 by forming different alkyl bonds (Table 6 and Figure 5(b)).

Dihydrocanaric acid showed binding affinity of -8.1 kcalmol-1 with COX-1 enzyme. Carboxyl group at C3 position formed strong conventional hydrogen bond with SER530 at a distance of 2.53 Å. Besides, different types of hydrophobic alkyl bond were formed with VAL349, ILE523, ALA527, LEU531, LEU359, LEU352, and ILE345. Different moieties of dihydrocanaric acid also interacted with TYR355 and PHE518 through the formation of four pi-alkyl bonds. Furthermore, the binding affinity of 15-bulnesolic acid and 1-(4-hydroxy-3-methoxyphenyl)-1-methoxypropan-2-ol were observed lower than that of the other compounds (Table 6 and Figures 5(c)–(e)).

#### 3.7.2. Molecular Docking of the Ligands with COX-2 Enzyme

**Figure 6:**
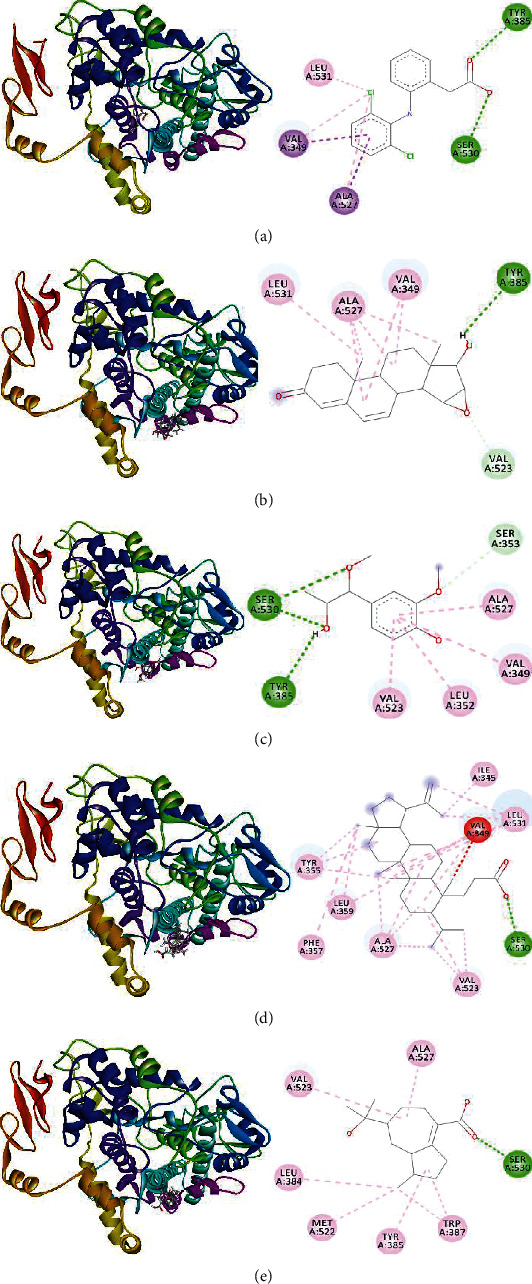
3D and 2D interaction of (a) diclofenac, (b) hoyasterone, (c) 1-(4-hydroxy-3-methoxyphenyl)-1-methoxypropan-2-ol, (d) dihydrocanaric acid, and (e) 15 bulnesolic acid, respectively, with COX-2 enzyme. Green, pink, light pink, and light green colors of the 2D image indicate hydrogen bond, Pi-Pi T shaped bond, Pi-alkyl bond, and carbon-hydrogen bond of that compound with COX-2.

Diclofenac showed binding affinity of -7.1 kcalmol-1 at the inhibitory site of COX-2. The carboxyl group of phenylyacetic acid moiety formed two separate conventional hydrogen bond with SER530 and TYR385 at a distance of 2.65 and 2.73 Å, respectively. Besides, (2,6-dichlorophenyl) amino moiety constituted one Pi-sigma bond and alkyl bond with VAL349. Moreover, another Pi-sigma bond together with alkyl bond was formed by this moiety with ALA527 and LEU531 (Table 6 and Figure 6(a)). Hoyasterone was found to be stable at the inhibitory site of COX-2 having binding affinity of -7.4 kcalmol-1. Hydroxyl group at C17 of hoyasterone formed a conventional hydrogen bond with the inhibitory motif TYR385 at 3.06 Å. Besides, C18 constituted two separate alkyl bonds by the interaction with LEU531 at 3.86 Å and ALA527 whereas C19 formed one alkyl bond through the interaction with ALA527. In addition, second ring and third ring of hoyasterone made a pair of alkyl bond with ALA527 and VAL349, respectively, (Table 6 and Figure 6(b)). The binding affinity of dihydrocanaric acid was calculated to be -7.2 kcalmol-1 at the inhibitory site of COX-2. The carboxyl group at C3 position formed conventional hydrogen bonds at 2.02 Å with SER530, another inhibitory motif of COX-2. Moreover, different types of alkyl bond were formed with ILE345, LEU531, VAL349, VAL523, ALA527, and LEU359. Additionally, dihydrocanaric acid formed four pi-alkyl bonds with TYR355 and PHE357, respectively. 15-bulnesolic acid and 1-(4-hydroxy-3-methoxyphenyl)-1-methoxypropan-2-ol showed binding affinity of -6.5 and -5.5 kcalmol^−1^ with COX-2 (Table 6 and Figures 6(c)–(e)).

### 3.8. ADMET (Absorption, Distribution, Metabolism, Excretion, and Toxicity) and Drug-Likeliness of Ligands

From the predicted data, we found that hoyasterone, 15-bulnesolic acid, and 1-(4-hydroxy-3-methoxyphenyl)-1-methoxypropan-2-ol were less lipophilic than diclofenac but dihydrocanaric acid showed highest lipophilic properties. Besides, gastro intestinal absorption, blood brain permeability, hepatotoxicity, Lipinski's rule of five violations and drug likeness of hoyasterone, 15-bulnesolic acid, and 1-(4-hydroxy-3-methoxyphenyl)-1-methoxypropan-2-ol were similar to diclofenac except dihydrocanaric acid. On the other hand, none of the compounds has any AMES toxicity [Table 7].

## 4. Discussion

Many products derived from medicinal plants are used in traditional medical systems for their medicinal properties, but some of them are being studied toxicologically. Therefore, toxicological studies should be conducted. Additionally, the amount of toxicity data for many plants is limited. Even in this case, pretoxicological data of plant-derived compounds can be found at a single acute toxicity dose. So, acute oral toxicity trials are crucial not only to recognize the adverse effects of a compound but also to estimate a therapeutic range of dose for continuous use. Maximum dose (4000 mg/kg) of the plant extracts (MHP, EAHP, and NHP) used in the acute toxicity study did not result in any sign of mortality for laboratory animals. Therefore, selected two doses (200 mg/kg and 400 mg/kg) of the plant extracts were considered safe with a wide range of therapeutic responses and were used in *in vivo* trials [35, 36].

Phenolic compounds are the most prevalent secondary metabolites and are extensively distributed across the plant kingdom. Antioxidant properties have been related to phenolic compounds and flavonoids, which function as scavengers of singlet oxygen and free radicals [61, 62]. The scavenging capability of the phenols is attributed to the hydroxyl groups in their chemical structure [63]. Flavonoids are secondary metabolites having antioxidant properties, the effectiveness of which is determined by the number and location of free OH groups [64]. The chemical structure of flavonoids governs their ability to inhibit free radical-mediated processes. Flavonoids have been found to be very efficient scavengers of most oxidizing chemicals, including singlet oxygen and other free radicals [65]. Flavonoids may affect both the propagation and formation of free radicals, either by chelating the transition metal or blocking the enzymes involved in the initiation step [66]. Antioxidant properties of tannins may be attributed to their ability to scavenge free radicals [67]. Tannins have the capacity to chelate metal ions, such as Fe(II), and prevent oxidation by interfering with one of the Fenton reaction steps [68]. In the present study, maximum amount of total phenolic compound was found to be 253.10 ± 1.56 mg GAE/g dry extract in MHP compared to EAHP and NHP. Hence, the presence of phenolic components in fractionated extracts of *H. parasitica* leaves indicates substantial antioxidant activity. In total flavonoid content assay, it is observed that MHP contained the highest amount of TFC (197.72 ± 1.03 mg QE/g dry extract) compared to EAHP and NHP due to the solvent polarity index. Besides, MHP disclosed the presence of the highest amount of TTC (112.70 ± 1.16 mg GAE/g dry extract) compared to EAHP and NHP.

A wide range of methods are used for determining the radical scavenging properties of antioxidants, and among them, DPPH is more preferable because of its high speed, flexibility, and reliability [69, 70]. Free radical scavenging activities of plant extracts depend on the ability of antioxidant compounds to diminish hydrogen atoms and structural conformation of these compounds [71]. Due to their ability to donate hydrogen, polyphenols and tocopherols scavenge DPPH radicals. The quantity of total polyphenols is firmly related to the free radical scavenging capability of antioxidants [72, 73]. In this assay, all extracts, namely, MHP, EAHP, and NHP, demonstrated concentration-dependent DPPH scavenging activity compared to that of ascorbic acid (IC_50_ 13.562 *μ*g/mL) where potent scavenging activity was revealed by MHP (70.890 *μ*g/mL) which may be due to presence of greater amount of polyphenols (Figure 1). Compounds that possess reducing power can act as primary or secondary antioxidant by inhibiting lipid peroxidation process [74]. A compound with reducing power has the ability to transfer electrons during redox reactions and thus convert free radicals to less reactive or inert products. However, buffer systems may improve antioxidant activity by modulating the ratio of protonated and deprotonated forms of antioxidants in addition to stabilizing the radical cation [75]. Numerous studies have examined the association between polyphenol structure and ferric reduction capacity [76, 77]. Fractionated extracts showed antioxidant activity in a concentration-dependent manner in reducing power experiment. Among the extracts, highest concentration of MHP and standard BHT (500 *μ*g/mL) showed maximum absorbance of 0.833 and 0.853, respectively (Figure 2).

The acetic acid-induced writhing test has been validated as a technique for assessing both central and peripheral antinociceptive activity [44]. Furthermore, the release of endogenous pain mediators such as prostaglandins and bradykinins. is implicated in the development of the writhing response as a model of visceral pain. The injection of acetic acid via the intraperitoneal route induces a rise in cyclooxygenase (COX), prostaglandins (PGs), serotonin, histamine, bradykinin, lipoxygenase (LOX), substance P, TNF-*α*, IL-1, and IL-8 in the peripheral tissue fluid. In response to elevated levels of those mediators, the primary afferent nociceptors are then incited to enter into the dorsal horn of the central nervous system. The release of these inflammatory mediators may result in increased permeability or breakdown of the blood-brain barrier (BBB). Again, intraperitoneal acetic acid (pain-inducing substance) injection also contributes to the increase of vasodilatation and permeability of vascular fluid [78]. Hence, antinociceptive effects of MHP, EAHP, and NHP might be achieved by either inhibiting the release of endogenous nociceptive mediators or by inhibiting the penetrability of both BBB and vascular fluid levels [78]. One of the most prominent acute pain models is the tail immersion model. In general, centrally functioning analgesics (opioids) are particularly associated with the tail-withdrawal response, whereas peripherally acting analgesics are not sensitive to heat-induced pain perception. Tail immersion is involved in spinal reflexes that act through the opioid receptors *μ*_2_ and *δ*. Furthermore, antinociceptive activity is elicited by centrally acting analgesics (opioids) in both early and late phases. The hot plate test is performed to figure out how centrally acting analgesics work. In this test, the supraspinal reflex is mediated by opioid receptors *μ*_1_ and *μ*_2_ [79]. The latency response of mice to heat stimulation is determined using the hot plate test. These plant extracts (MHP, EAHP, and NHP) may exert antinociceptive effects in the central nervous system by stimulating periaqueductal gray matter (PAG), which releases endogenous peptides like endorphin and enkephalin. These endogenous peptides descend the spinal cord and block the transmission of pain impulses at the synaptic junction of the dorsal horn [1].

The majority of antidiarrheal drugs work by lowering GI (gastrointestinal) secretion or shortening GI smooth muscle propulsive action. The castor oil-driven diarrheal model is commonly used to assess antidiarrheal efficacy in secretory and motility diarrhea [80]. Castor oil limits the reabsorption of water into the gastrointestinal tract and increases the volume of intestinal content and generates diarrhea. Ricinoleic acid is an active metabolite of castor oil that stimulates prostaglandin release and causes irritation and inflammation in the intestinal mucosa. Prostaglandins increase gastrointestinal secretion by preventing sodium chloride and water from being reabsorbed [81]. Increased PGE2 compositions in the gut lumen enhance the net release of water and electrolytes into the small intestine [82]. Ricinoleic acid interacts with both Na+ and K+ in the intestinal lumen to generate ricinoleate. On the mucosa, ricinoleate has an anti-absorptive action. Ricinoleate inhibits the action of Na+, K+ ATPase, leading the intestinal epithelium to be more permeable. Castor oil-induced diarrhea can be delayed through the use of prostaglandin synthesis inhibitors [83]. Magnesium sulfate-induced diarrheal test is a common and widely used model for evaluating antidiarrheal activity. Oral ingestion of MgSO_4_ increases the secretion and motility of the small intestine by promoting the secretion of cholecystokinin and induces diarrhea. Cholecystokinin has two key functions; firstly, it stimulates secretion and motility in the small intestine, and secondly, it inhibits the absorption of sodium chloride and water. It causes fluid accumulation in the intestinal lumen as well as movement (from proximal to the distal intestine) [84, 85]. As a result of anti-electrolyte permeability action, the extracts (MHP, EAHP, and NHP) may have antidiarrheal properties against castor oil as well as magnesium sulfate induced diarrhea [86].

In animal models, exogenous substances such as bacterial endotoxins and microbial infection may produce fever. Exogenous pyrogen promotes the release of proinflammatory cytokines such as TNF-*α*, IL-1*β*, IFN-*α*, and IL-6 into the hypothalamus circulation as well as the release of local PGs and PGE2 which raises the body's temperature [87]. Flavonoids and alkaloids retard the generation of PG which might be responsible for antipyretic activity of the plant extracts (MHP, EAHP, and NHP, respectively) [88, 89].

Virtual screening plays a vital role in finding conceptually active compounds which have been formerly identified but not subjected for any bioactivity against particular drug targets together with desired ADMET properties. This anticipation process precedes to lead discovery beyond the random screening [90]. The topological polar surface area is considered to be a significant parameter for the easy access of compounds to the cell membranes [91].

In general, the drug molecules exert specific pharmacological response by interacting with protein structure which act as a target for them. 3D structure of the interested protein is obtained through the utilization of particular software as well as online protein data bank (PDB) in molecular docking approach. The possible interaction of the ligands with interested protein is determined based on the binding energies of the ligands [90]. In molecular docking studies, there exist two significant factors, namely, affinity values and type of interaction. The determination of protein-ligand sites is linked to hydrogen bond, electrostatic potential, and Van der Walls interaction [92]. Hence, *in silico* study along with *in vitro* and *in vivo* studies was carried out in the present study because of the major contribution in the drug development process. The identified compounds of *H. parasitica* were docked against COX-1 and COX-2 as these enzymes have been reported for regulating analgesic and antipyretic activities. Hoyasterone under androstanoid class not only showed strong hydrogen bond with inhibitory motif TYP385 but also higher binding affinity to the inhibitory site of COX-1 and COX-2 than the rest of the ligands. All of the compounds revealed drug-like properties according to Lipinski's rule and also showed prominent binding affinity to the active site of the enzymes. Moreover, they revealed several conventional hydrogen bonds to the enzymes. Therefore, binding affinity data and interaction patterns of the compounds of *H. parasitica* revealed that they can contribute to treat pain and pyresis. Among the compounds, hoyasterone can be a promising lead compound to inhibit COX-1 and COX-2 enzymes.

## 5. Conclusion

The present study disclosed the analgesic activity of fractionated extracts of *H. parasitica* leaves in both central and peripheral pain models along with antidiarrheal and antipyretic activities. Besides, among 4 different isolated compounds, hoyasterone was found to be prominent lead compound interacting with cyclooxygenase enzymes. Again, ADME/toxicity analysis showed acceptable pharmacokinetics and toxicity profiles of hoyasterone. Therefore, isolation of bioactive compound responsible for analgesic and antipyretic properties is inevitable for the elucidation of exact mechanism.

## Figures and Tables

**Table 1:** Qualitative phytochemical screening of NHP, EAHP, and MHP extracts.

Phytochemical group	NHP	EAHP	MHP
Carbohydrate	+	+	+
Alkaloid	+	+	+
Phenolic compound	—	+	+
Flavonoids	+	+	+
Tannins	+	+	+
Saponins	—	—	—
Glycosides	+	+	+
Anthraquinone	+	—	—
Steroids	+	—	—
Gums	+	+	+
Acidic compounds	—	+	+

Here, “+” indicates present and “–—” indicates absent.

**Table 2:** Effect of MHP, EAHP, and NHP in the acetic acid-induced writhing test.

Group	Dose	Number of writhing	Inhibition (%)
Control	10 ml/kg	43.20 ± 1.02	0.00 ± 0.00
DS	25 mg/kg	6.20 ± 0.73^∗^^■▲◊♯*Δ*^	85.77 ± 1.39^∗^^■▲◊♯*Δ*^
MHP	200 mg/kg	16.20 ± 0.58^∗^^*θ*▲♯*Δ*^	62.49 ± 0.67^∗^^*θ*▲♯*Δ*^
MHP	400 mg/kg	11.20 ± 0.66^∗^^▲◊♯*Δ*^	74.15 ± 1.00^∗^^▲◊♯*Δ*^
EAHP	200 mg/kg	26.00 ± 1.41^∗^^*θ*■□♯*Δ*^	39.80 ± 2.94^∗^^*θ*■□♯*Δ*^
EAHP	400 mg/kg	21.00 ± 1.52^∗^^*θ*□♯*Δ*^	51.52 ± 2.80^∗^^*θ*□♯*Δ*^
NHP	200 mg/kg	35.00 ± 1.90^∗^^*θ*■□▲◊^	18.50 ± 5.89^∗^^*θ*■□▲◊^
NHP	400 mg/kg	32.00 ± 1.30^∗^^*θ*■□▲◊^	25.80 ± 3.29^∗^^*θ*■□▲◊^

Values are presented as mean ± standard error of mean. *n* =5 mice in each group. ^∗^*P* < 0.05, versus control (Dunnett's t test); ^*θ*^*P* < 0.05, versus DS 25 mg/kg; ^■^*P* < 0.05, versus MHP 200 mg/kg; ^□^*P* < 0.05, versus MHP 400 mg/kg;^▲^*P* < 0.05, versus EAHP 200 mg/kg; ^◊^*P* < 0.05, versus EAHP 400 mg/kg; ^♯^*P* < 0.05, versus NHP 200 mg/kg;^Δ^*P* < 0.05, versus NHP 400 mg/kg (pair-wise comparison by post hoc Tukey's HSD test).

**Table 3:** Effects of standard, MHP, EAHP, and NHP in tail immersion test.

Group	Dose	Latency time (S)				
		0 min	30 min	60 min	120 min	180 min
Control	10 ml/kg	1.50 ± 0.8	1.84 ± 0.07	2.16 ± 0.06	2.30 ± 0.02	1.41 ± 0.08
Tramadol	10 mg/kg	3.09 ± 0.30^∗^^■□▲◊♯*Δ*^	4.06 ± 0.09^∗^^■□▲◊♯*Δ*^	5.33 ± 0.25^∗^^■□▲◊♯*Δ*^	7.28 ± 0.17^∗^^■□▲◊♯*Δ*^	4.52 ± 0.23^∗^^▲◊♯*Δ*^
MHP	200 mg/kg	2.04 ± 0.12^∗^*^θ^*	2.66 ± 0.13^∗^^*θ*▲♯*Δ*^	3.10 ± 0.14^∗^^*θ*▲◊♯*Δ*^	2.31 ± 0.14^*θ*□◊♯^	4.21 ± 0.11^∗^^▲◊♯*Δ*^
MHP	400 mg/kg	2.26 ± 0.22^∗^^*θ*♯^	2.36 ± 0.13^∗^^*θ*♯*Δ*^	3.49 ± 0.16^∗^^*θ*▲◊♯*Δ*^	3.21 ± 0.14^∗^^*θ*■▲♯*Δ*^	4.52 ± 0.16^∗^^▲◊♯^
EAHP	200 mg/kg	1.90 ± 0.11*^θ^*	1.98 ± 0.07^*θ*■^	2.11 ± 0.03^*θ*■□^	1.89 ± 0.09^*θ*□◊^	2.22 ± 0.18^∗^^*θ*■□^
EAHP	400 mg/kg	2.05 ± 0.07^∗^*^θ^*	2.27 ± 0.13^∗^^*θ*♯^	2.20 ± 0.07^*θ*■□^	2.84 ± 0.06^∗^^*θ*■▲♯^	2.76 ± 0.10^∗^^*θ*■□♯*Δ*^
NHP	200 mg/kg	1.47 ± 0.09^*θ*□^	1.59 ± 0.07^*θ*■□◊^	2.15 ± 0.04^*θ*■□^	1.51 ± 0.08^∗^^*θ*■□◊*Δ*^	2.05 ± 0.09^∗^^*θ*■□◊^
NHP	400 mg/kg	1.75 ± 0.07*^θ^*	1.84 ± 0.12^*θ*■□^	2.48 ± 0.12^*θ*■□^	2.31 ± 0.14^*θ*□♯^	1.97 ± 0.11^∗^^*θ*■□◊^

Values are presented as mean ± standard error of mean. *n* =5 mice in each group. ^∗^*P* < 0.05, versus control (Dunnett's t test); ^*θ*^*P* < 0.05, versus tramadol 10 mg/kg; ^■^*P* < 0.05, versus MHP 200 mg/kg; ^□^*P* < 0.05, versus MHP 400 mg/kg; ^▲^*P* < 0.05, versus EAHP 200 mg/kg; ^◊^*P* < 0.05, versus EAHP 400 mg/kg; ^♯^*P* < 0.05, versus NHP 200 mg/kg; ^Δ^*P* < 0.05, versus NHP 400 mg/kg (pair-wise comparison by post hoc Tukey's HSD test).

**Table 4:** Effects of MHP, EAHP, and NHP in Hot plate test.

Group	Dose	Latency time (S)				
		0 min	30 min	60 min	120 min	180 min
Control	10 ml/kg	2.47 ± 0.10	2.62 ± 0.08	2.55 ± 0.11	2.36 ± 0.10	2.76 ± 0.13
Tramadol	10 mg/kg	4.60 ± 0.13^∗^^■□▲♯*Δ*^	5.84 ± 0.09^∗^^■□▲◊♯*Δ*^	5.97 ± 0.04^∗^^■□▲◊♯*Δ*^	6.29 ± 0.12^∗^^■□▲◊♯^	7.40 ± 0.25^∗^^■□▲◊♯*Δ*^
MHP	200 mg/kg	3.78 ± 0.07^∗^^*θ*♯*Δ*^	3.95 ± 0.09^∗^^*θ*▲◊♯*Δ*^	4.22 ± 0.14^∗^^*θ*▲♯*Δ*^	4.15 ± 0.10^∗^^*θ*□◊♯^	3.66 ± 0.16^∗^^*θ*□◊♯^
MHP	400 mg/kg	3.43 ± 0.14^∗^^*θ*◊♯*Δ*^	3.67 ± 0.12^∗^^*θ*▲◊♯*Δ*^	4.50 ± 0.13^∗^^*θ*▲♯*Δ*^	5.72 ± 0.21^∗^^*θ*■▲♯^	5.88 ± 0.16^∗^^*θ*■▲♯*Δ*^
EAHP	200 mg/kg	3.37 ± 0.09^∗^^*θ*◊♯*Δ*^	3.15 ± 0.13^∗^^*θ*■□◊♯^	3.58 ± 0.20^∗^^*θ*■□◊♯^	4.09 ± 0.10^∗^^*θ*□◊♯^	3.95 ± 0.06^∗^^*θ*□◊♯*Δ*^
EAHP	400 mg/kg	4.15 ± 0.10^∗^^□▲♯*Δ*^	4.47 ± 0.12^∗^^*θ*■□▲♯*Δ*^	4.30 ± 0.14^∗^^*θ*▲♯*Δ*^	5.20 ± 0.08^∗^^*θ*■▲♯^	5.53 ± 0.19^∗^^*θ*■▲♯*Δ*^
NHP	200 mg/kg	2.30 ± 0.16^*θ*■□▲◊^	2.37 ± 0.70^■□▲◊*Δ*^	2.83 ± 0.08^*θ*■□▲◊^	3.08 ± 0.06^*θ*■□▲◊^	2.24 ± 0.10^*θ*■□▲◊*Δ*^
NHP	400 mg/kg	2.17 ± 0.07^*θ*■□▲◊^	2.95 ± 0.08^■□◊♯^	3.24 ± 0.08^∗^^*θ*■□◊^	3.43 ± 0.07^∗^^*θ*■□▲◊^	3.10 ± 0.06^*θ*□▲◊♯^

Values are presented as mean ± standard error of mean. *n* =5 mice in each group. ^∗^*P* < 0.05, versus control (Dunnett's t test); ^*θ*^*P* < 0.05, versus tramadol 10 mg/kg; ^■^*P* < 0.05, versus MHP 200 mg/kg; ^□^*P* < 0.05, versus MHP 400 mg/kg; ^▲^*P* < 0.05, versus EAHP 200 mg/kg; ^◊^*P* < 0.05, versus EAHP 400 mg/kg; ^♯^*P* < 0.05, versus NHP 200 mg/kg; ^Δ^*P* < 0.05, versus NHP 400 mg/kg (pair-wise comparison by post hoc Tukey's HSD test).

**Table 5:** Effects of MHP, EAHP, and NHP in brewer's yeast-induced pyrexia test.

Group	Dose	Initial rectal temperature (°C)	Rectal temperature after 18 h of yeast injection (°C)				
			0 h	1 h	2 h	3 h	4 h
Control	10 ml/kg	37.33 ± 0.22	38.22 ± 0.16	38.44 ± 0.18	38.54 ± 0.28	38.15 ± 0.13	38.63 ± 0.20
Paracetamol	100 mg/kg	36.67 ± 0.21^∗^^◊♯^	37.53 ± 0.15^■^	37.15 ± 0.09^∗^^■◊♯^	36.87 ± 0.09^∗^^■▲◊♯^	35.72 ± 0.25^∗^^■□▲◊♯*Δ*^	35.25 ± 0.18^∗^^■▲◊♯*Δ*^
MHP	200 mg/kg	37.25 ± 0.11^□^	39.43 ± 0.19^∗^^*θ*□▲◊♯*Δ*^	38.94 ± 0.07^*θ*□▲◊♯*Δ*^	38.33 ± 0.19^*θ*□*Δ*^	37.15 ± 0.10^∗^^*θ*♯^	37.38 ± 0.16^∗^^*θ*□♯^
MHP	400 mg/kg	36.16 ± 0.16^∗^^■▲◊♯*Δ*^	37.44 ± 0.16^∗^^■^	37.25 ± 0.16^∗^^■♯^	36.83 ± 0.12^∗^^■▲◊♯^	36.95 ± 0.16^∗^^*θ*♯^	35.76 ± 0.34^∗^^■◊♯*Δ*^
EAHP	200 mg/kg	37.12 ± 0.16^□^	37.98 ± 0.28^■^	37.73 ± 0.15^∗^^■^	37.88 ± 0.13^∗^^*θ*□^	36.64 ± 0.21^∗^^*θ*♯^	36.52 ± 0.17^∗^^*θ*♯^
EAHP	400 mg/kg	37.62 ± 0.08^*θ*□*Δ*^	38.10 ± 0.19^■^	37.93 ± 0.15^*θ*■^	37.98 ± 0.07^*θ*□*Δ*^	37.43 ± 0.21^∗^*^θ^*	37.19 ± 0.17^∗^^*θ*□♯^
NHP	200 mg/kg	36.90 ± 0.13^*θ*□^	38.22 ± 0.16^■^	38.19 ± 0.15^*θ*■□*Δ*^	38.27 ± 0.17^*θ*□*Δ*^	38.13 ± 0.14^*θ*■□▲*Δ*^	38.30 ± 0.13^*θ*■□▲◊*Δ*^
NHP	400 mg/kg	37.07 ± 0.09^□◊^	37.63 ± 0.09^■^	37.41 ± 0.22^∗^^■♯^	37.15 ± 0.12^∗^^■◊♯^	37.24 ± 0.13^∗^^*θ*♯^	36.82 ± 0.14^∗^^*θ*□♯^

Values of rectal temperature (°C) are displayed as mean ± standard error of mean. *n* =5 mice in each group. ^∗^*P* < 0.05, vs. control (Dunnett's t test); ^*θ*^*P* < 0.05, vs. paracetamol 100 mg/kg; ^■^*P* < 0.05, vs. MHP 200 mg/kg; ^□^*P* < 0.05, vs. MHP 400 mg/kg; ^▲^*P* < 0.05, vs. EAHP 200 mg/kg; ^◊^*P* < 0.05, vs. EAHP 400 mg/kg;^♯^*P* < 0.05, vs. NHP 200 mg/kg; ^Δ^*P* < 0.05, vs. NHP 400 mg/kg (pair-wise comparison by post hoc Tukey's HSD test).

**Table 6:** Molecular interaction of the ligands with COX-1 and COX-2.

Isoforms	Compounds	Binding affinity	Interaction with amino acids	
			Hydrogen bonds	Hydrophobic bonds
COX-1	Diclofenac	-8.1	TYR385 (2.68 Å); SER530 (2.53 Å)	ALA527; LEU352; VAL349; LEU531; and ILE523
	Hoyasterone (101449264)	-8.5	TYR385 (2.78 Å)	VAL349; ILE523; ALA527; and LEU531
	Dihydrocanaric acid (101449266)	-8.1	SER530 (2.53 Å)	VAL349; ILE523; ALA527; LEU531; LEU359; LEU352; ILE345; TYR355; and PHE518
	15-bulnesolic acid	-6.5	TYR385 (2.57 Å)	VAL349; ALA527; LEU352; ILE523; and TRP387
	1-(4-hydroxy-3-methoxyphenyl)-1-methoxypropan-2-ol. (78152111)	-6.4	VAL 349 (3.07 Å); TYR385(2.17 Å); MET522 (3.45 Å); and SER530 (3.25 Å)	VAL349; and LEU352
COX-2	Diclofenac	-7.1	TYR385 (2.73 Å); SER530 (2.65 Å)	VAL349; ALA527; and LEU531
	Hoyasterone (101449264)	-7.4	TYR385 (3.06 Å); VAL523 (3.59 Å)	VAL349; ALA527; and LEU531
	1-(4-hydroxy-3-methoxyphenyl)-1-methoxypropan-2-ol. (78152111)	-5.5	SER530 (2.42 Å); TYR385(2.58 Å); SER353(3.48 Å)	VAL349; VAL523; LEU352; and ALA527
	Dihydrocanaric acid (101449266)	-7.2	SER 530 (2.02 Å)	VAL349; LEU359; VAL523; ALA527; LEU531; ILE345; TYR355; and PHE357
	15-bulnesolic acid	-6.5	SER530 (1.94 Å)	VAL523; ALA527; LEU384; MET522; TYR385; and TRP387

**Table 7:** ADMET analysis of ligands.

S.I.	Compounds	MW	NHA	NHD	LogP	NRB	GIA	LD50	BBB	HT	AT	NLV	DL
1	Diclofenac	296.15	2	2	4.3641	4	High	2.405	Yes	No	No	0	Yes
2	Hoyasterone	300.398	3	1	2.6424	0	High	2.066	Yes	No	No	0	Yes
3	1-(4-hydroxy-3-methoxyphenyl)-1-methoxypropan-2-ol.	212.245	4	4	1.4691	2	High	2.064	Yes	No	No	0	Yes
4	Dihydrocanaric acid	442.728	1	1	8.3647	5	Low	2.628	No	Yes	No	1	Yes
5	15-bulnesolic acid	252.354	2	2	1.696	2	High	1.696	Yes	No	No	0	Yes

MW: molecular weight (g/mol); NHA: no. of hydrogen bond acceptor; NHD: no. of hydrogen bond donor; LogP: predicted octanol/water partition coefficient; NRB: number of rotatable bond; GIA: Gastro Intestinal absorption; LD50: median lethal dose; BBB: blood brain barrier; HT hepatotoxicity; AT: AMES toxicity; NLV: number of Lipinski's Violation; DL: drug likeness.

## Data Availability

Only the summary of the data has been reported in this article. On the basis of reasonable request, datasets are available from the corresponding author.

## Abbreviations

MHP: Methanol extract of *Hoya parasitica* Wall. leaves
EAHP: Ethyl acetate extract of *Hoya parasitica* Wall. leaves
NHP: N-hexane extract of *Hoya parasitica* Wall. leaves
COX 1: Cyclooxygenase 1
COX 2: Cyclooxygenase 2
DPPH: 2,2-diphenyl-1-picrylhydrazyl
TPC: Total phenolic content
TFC: Total flavonoid content
TTC: Total tannin content
OECD: Organization of Economic Cooperation and Development
LD_50_: Median lethal dose
DMSO: Dimethyl sulfoxide
BHT: Butylated hydroxy tolune
TNF-*α*: Tumor necrosis factor-*α*
IL-1: Interleukin-1
IL-6: Interleukin-6
NSAIDs: Non-steroidal anti-inflammatory drugs
PGs: Prostaglandins
ADMET: Absorption, distribution, metabolism, excretion, and toxicity
MW: Molecular weight (g/mol)
NHA: No. of hydrogen bond acceptor
NHD: No. of hydrogen bond donor
LogP: Predicted octanol/water partition coefficient
NRB: Number of rotatable bond
GIA: Gastro intestinal absorption
BBB: Blood brain barrier
HT: Hepatotoxicity
AT: AMES toxicity
NLV: Number of Lipinski's violation
DL: Drug likeness.
